# The phylogeny of *Seseli* (Apiaceae, Apioideae): insights from molecular and morphological data

**DOI:** 10.1186/s12870-022-03919-9

**Published:** 2022-11-16

**Authors:** Jing Cai, Huan-Huan Qin, Jia-Qing Lei, Chang-Kun Liu, Xing-Jin He, Song-Dong Zhou

**Affiliations:** Key Laboratory of Bio-Resources and Eco-Environment of Ministry of Education, College of Life Sciences, Sichuan University, Chengdu, 610065 China

**Keywords:** Apiaceae, *Seseli*, Plastome, Phylogeny, Morphology, Taxonomy

## Abstract

**Background:**

The genus *Seseli* L., which consists of 125–140 species distributed in the Old World from western Europe and northwestern Africa to China and Japan, is one of the largest and most taxonomically difficult genera of Apiaceae Lindl. Although several previous studies have been conducted on *Seseli* based on limited morphological characteristics and molecular fragments, a robust and comprehensive phylogeny of *Seseli* remains elusive. Plastomes provide abundant genetic information and have been widely used in studying plant phylogeny and evolution. Consequently, we newly generated the complete plastomes of eleven *Seseli* taxa. We combined plastome data and morphological characteristics to investigate the phylogeny of *Seseli*.

**Results:**

In our study, we observed that the genome length, gene numbers, IR/SC borders, and repeat composition of the eleven *Seseli* plastomes were variable. Several appropriate mutation hotspot regions may be developed as candidate DNA barcodes for evolution, phylogeny, and species identification of *Seseli*. The phylogenetic results identified that *Seseli* was not a monophyletic group. Moreover, the eleven newly sequenced *Seseli* taxa did not cluster with *S. tortuosum* (the type species of *Seseli*, belonging to the tribe Selineae), where *S. delavayi* clustered with *Eriocycla* belonging to the tribe Echinophoreae and the other ten belonged to Selineae. The comparative plastome and morphological characteristics analyses confirmed the reliability of the phylogenetic analyses and implied the complex evolution of *Seseli*.

**Conclusion:**

Combining molecular and morphological data is efficient and useful for studying the phylogeny of *Seseli*. We suggest that “a narrow sense” of *Seseli* will be meaningful for further study and the current taxonomic system of *Seseli* needs to be revised. In summary, our study can provide new insights into the phylogenetic relationships and taxonomic framework of *Seseli*.

**Supplementary Information:**

The online version contains supplementary material available at 10.1186/s12870-022-03919-9.

## Background

*Seseli* L. is one of the largest genera of Apiaceae Lindl. [1] and consists of 125–140 species. *Seseli* species are distributed in the Old World from western Europe and northwestern Africa to China and Japan [1–4]. Nineteen *Seseli* species are distributed in China with nine of them endemic [5].

As one of the largest genera of Apiaceae, the taxonomy of *Seseli* has been controversial so far. One of the prominent taxonomic problems is the inclusion of *Libanotis* Haller ex Zinn and *Eriocycla* Lindl. within *Seseli*. For *Libanotis*, Drude regarded *Libanotis* as one of the four subgenera of *Seseli* [6]. Then, Pimenov and Sdobnina classified *Libanotis* distributed in Russia into different groups within *Seseli* [7]. Pimenov summarized previous studies and type specimens, and concluded that most Chinese *Libanotis* species were synonyms of *Seseli* species [e.g., *Libanotis buchtormensis* (Fisch.) DC. $\equiv$ *Seseli buchtormense* (Spreng.) W. D. J. Koch, *Libanotis montana* Crantz $\equiv$ *Seseli libanotis* (L.) W. D. J. Koch] [8]. In the latest research, Duran et al*.* [9] found that species of *Libanotis* form a clade, but this clade was falling into polytomy with other *Seseli* species. However, Schischkin advocated the generic status of *Libanotis* according to its different morphological characteristics (e.g., conspicuous calyx teeth, bracts numerous, the separation of bracteoles, and almost always pubescent fruits) [10]. For *Eriocycla*, Kljuykov proposed that the type species of *Eriocycla* (*Eriocycla nuda* Lindl.) with its related species and several *Seseli* species should be divided into a new section of *Seseli* as there were no substantial carpological differences between *Eriocycla* and *Seseli* and were similar in non-carpological characteristics [11]. Moreover, Pimenov treated all *Eriocycla* species distributed in China as synonyms of *Seseli* species [e.g., *Eriocycla nuda* $\equiv$ *Seseli nudum* (Lindl.) Pimenov et Kljuykov, *Eriocycla pelliotii* (H. Boissieu) H. Wolff $\equiv$ *Seseli pelliotii* (H. Boissieu) Pimenov et Kljuykov] [8]. However, Degtjareva et al*.* proposed that several *Seseli* species [e.g., *S. delavay*i Franch., *S. afghanicum* Pimenov] should be transferred into *Eriocycla* [12]. In addition, taxonomic boundaries were uncertain between *Seseli* and *Ligusticum mucronatum* (Schrenk ex Fisch. & C.A. Mey.) Leute. For instance, Pimenov treated *Ligusticum mucronatum* and *Ligusticum thomsonii* C. B. Clarke as synonyms of *Seseli mucronatum* (Schrenk) Pimenov et Sdobnina [8]. Most of the above-mentioned taxonomic treatments were based on limited morphological data, but this was insufficient to define the boundaries between *Seseli* and its related genera. For example, fruit structures of the type species of *Seseli* (*S. tortuosum*) and *Libanotis* (*Libanotis montana* Crantz) were almost identical [13], and different types of fruit structures existed among *Seseli* species [14]. Therefore, combining more abundant morphological characteristics is critical to resolving the taxonomy of *Seseli*.

An ideal genus should be monophyletic and clearly defined based on morphology [15]. *Seseli* is not a monophyletic group, which is the same as other large genera of Apiaceae (e.g., *Angelica* L., *Ligusticum* L., *Peucedanum* L.) and is one of the most taxonomically complicated genera within Apiaceae [16–18]. Previous studies have used several molecular fragments (e.g., ITS, *rps16* intron, *rpl16* intron) to show that members belonging to *Seseli* are distributed into three tribes: Selineae (including the majority of *Seseli* species), Pimpinelleae [*S. diffusum* (Roxb. ex Sm.) Santapu & Wagh] and Apieae (*S. webbii* Coss.) [15, 17, 19–21]. However, these molecular fragments contained too few informative sites. Consequently, the results of these molecular studies showed low support and resolution and were insufficient to resolve the phylogeny of *Seseli*. Therefore, it is urgent to use extensive sampling and abundant molecular data to reconstruct a more robust phylogeny of *Seseli*.

*Seseli* is an important genus with a high number of aromatic species used as traditional medicine due to their richness in coumarins, terpenoids, and essential oils. They have many important pharmacological activities such as reduction of inflammation, swelling, rheumatism, pain, and minimization of the common cold’s symptoms [22–24]. Five *Seseli* taxa (*S. mairei* H. Wolff, *S. mairei* var. *simplicifolia* C. Y. Wu ex R. H. Shan & M. L. Sheh, *S. yunnanense* Franch., *S. delavayi* Franch., and *S. squarrulosum* Shan & M.L. Sheh) are used as traditional Chinese medicine “Fang feng” [5]. The identification of medicinal materials is almost entirely based on morphological characteristics and traditional recognition. However, medicinal materials are used indiscriminately given the considerable intraspecific morphological variations of *Seseli* species. Hence, it is necessary to define species boundaries and develop more molecular markers to ensure the correct identification and usage of medicinal *Seseli* species.

The length of a plastome is usually 115 kb-165 kb. The plastome’s typical quadripartite structure is composed of a pair of inverted repeat (IR) regions of 22-25 kb separating the large single-copy (LSC) region of 82-90 kb and the small single-copy (SSC) region of 15-20 kb [25]. Plastome of angiosperms has the advantages of low nucleotide substitution rates and without gene recombination [26]. Plastomes have been widely used in studying the phylogeny of Apiaceae, Ranunculaceae Juss., Saxifragaceae Juss., *Allium* L., Liliaceae Juss., etc. [27–37]. However, there has been no study on the phylogeny of *Seseli* based on plastomes and therefore there is ample opportunity to investigate phylogenetic and taxonomic issues of *Seseli* using plastomes.

Plastome is valuable for phylogenetic studies but should be combined with morphological characteristics (especially carpological characteristics) given the significance of morphology in the taxonomy and evolution of Apiaceae. For example, Wen et al*.* and Li et al*.* have combined molecular phylogenetic analyses with carpological characteristics to obtain relatively reliable results [29, 38]. Thus, we use plastomes and morphological data to explore the phylogeny of *Seseli*. Here, our aims were to: (1) investigate the plastome features and evolution of *Seseli*; (2) develop appropriate mutation hotspot regions as candidate DNA barcodes for species identification of *Seseli*; (3) test the ability of plastomes to study the phylogeny of *Seseli*; and (4) explore the effectiveness of comparative plastome analyses and morphological data for studying the phylogeny and taxonomy of *Seseli*. Overall, our study can provide new insights into the phylogenetic relationships and taxonomic framework of *Seseli*.

## Results

### Features of the eleven *Seseli* plastomes

**Fig. 1:**
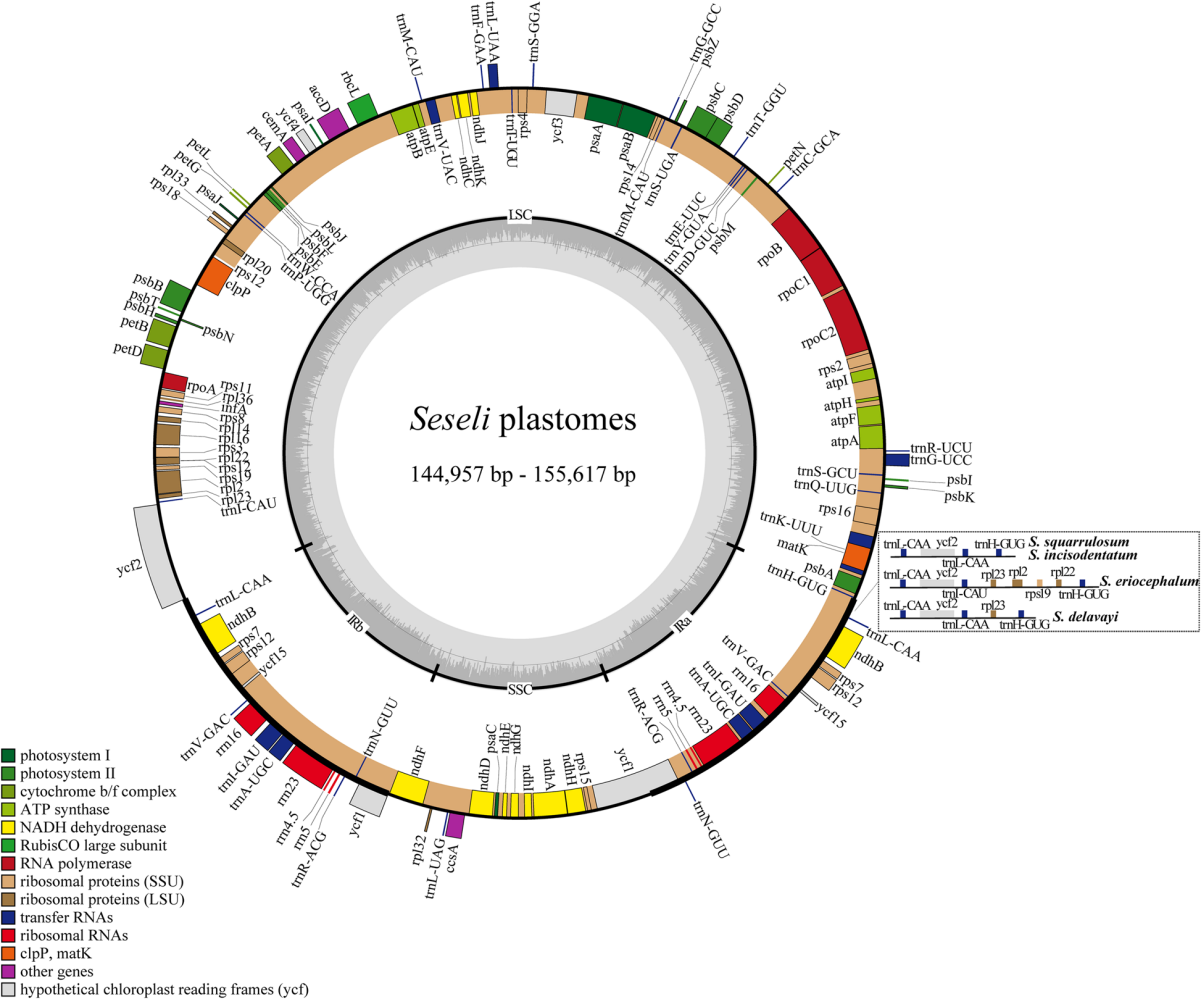
Gene map of the eleven *Seseli* plastomes. Genes shown outside and inside the outer layer circle are transcribed in the clockwise and counterclockwise, respectively. Different functional groups of genes are marked with different colors. The dark gray area of the inner circle represents the GC content of the plastome. IR, inverted repeat, LSC, large single copy, SSC, small single copy

**Fig. 2:**
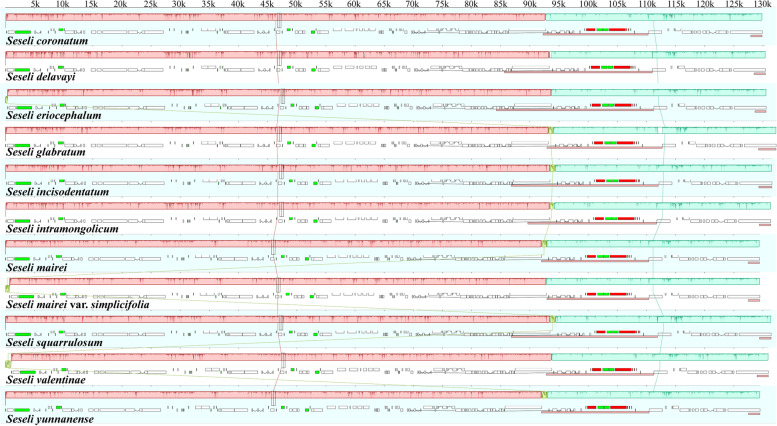
Mauve alignment of the eleven *Seseli* plastomes. Local collinear blocks within each alignment are represented by blocks of the same color connected with lines

The size of the eleven *Seseli* plastomes ranged from 144,957 bp in *S. mairei* var. *simplicifolia* to 155,617 bp in *S. eriocephalum* (Pall. ex Spreng.) Schischk. All eleven plastomes shared the typical quadripartite structure consisting of a pair of IRs (17,473-26,992 bp) separating the large single copy (LSC) region (84,243-92,935) and the small single copy (SSC) region (16,501-17,698 bp) (Fig. 1, Table 1). The overall GC content ranged from 37.4% to 37.6%, while the IR regions were 42.5-44.9% and much higher than the LSC regions (35.6-36.0%) and SSC regions (30.8-31.2%). In addition, the eleven plastomes contained 128-134 genes, including 84-89 protein-coding genes, 36-37 transfer RNA (tRNA) genes, and eight ribosomal RNA (rRNA) genes (Table 1). No gene rearrangement or loss was found in the eleven *Seseli* plastomes (Fig. 2, Table S3).

**Table 1:** Features of the eleven *Seseli* plastomes

Taxa	Total length (bp)				GC content (%)				Gene numbers			
	Size	LSC	SSC	IR	Total	LSC	SSC	IR	Total	Protein-coding genes	tRNA	rRNA
*S. coronatum*	145,937	92,284	17,603	18,025	37.6	36.0	30.9	44.7	128	84	36	8
*S. delavayi*	153,859	85,660	17,427	25,386	37.6	35.8	31.1	42.8	131	86	37	8
*S. eriocephalum*	155,617	84,243	17,390	26,992	37.6	35.8	31.2	42.5	134	89	37	8
*S. glabratum*	149,039	92,922	16,501	19,808	37.5	36.0	31.2	43.7	128	84	36	8
*S. incisodentatum*	154,590	86,792	17,208	25,295	37.4	35.7	30.8	42.6	130	85	37	8
*S. intramongolicum*	151,526	89,654	17,592	22,140	37.6	35.8	31.0	43.7	128	84	36	8
*S. mairei*	145,859	91,984	17,061	18,407	37.5	36.0	31.0	44.3	128	84	36	8
*S. mairei* var. *simplicifolia*	144,957	92,935	17,076	17,473	37.5	35.9	31.0	44.9	128	84	36	8
*S. squarrulosum*	154,502	86,815	17,485	25,101	37.4	35.6	30.8	42.7	130	85	37	8
*S. valentinae*	147,460	92,816	17,698	18,473	37.5	35.9	30.9	44.7	128	84	36	8
*S. yunnanense*	145,975	92,014	17,067	18,447	37.5	35.9	31.1	44.3	128	84	36	8

### Repeat sequence and nucleotide diversity analyses

**Fig. 3:**
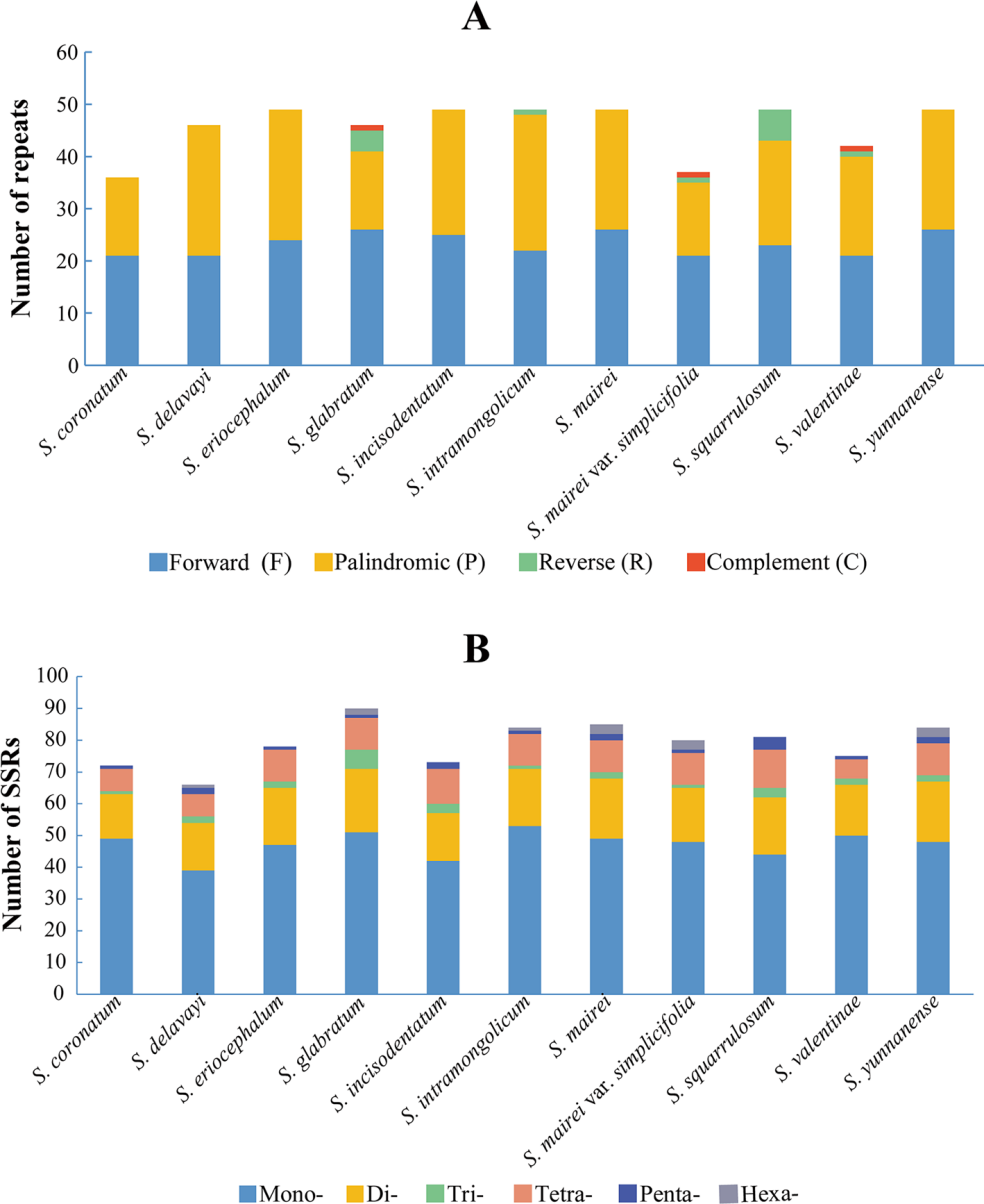
Analyses of repeats in the eleven *Seseli* plastomes. (**A**) Total number of four repeat types, (**B**) Total number of SSRs

In the eleven *Seseli* plastomes, we found 501 repeats of four types, with the number of repeats varying from 36 to 49 between species (Fig. 3A, Table S4). The most abundant repeats were forward repeats (256), followed by palindromic repeats (229), reverse repeats (13), and the least were complementary repeats (3), which only appeared in three taxa (*S. glabratum* Willd. ex Schult., *S. mairei* var. *simplicifolia*, and *S. valentinae* Popov.) (Fig. 3A). Most of the repeats were distributed in intergenic or intron regions (e.g., *ycf2*-*trnL*-*CAA*, *trnL*-*CAA-trnH*-*GUG*, *ycf3* intron, *ndhA* intron). However, protein-coding genes also contained a few repeats, among which the *ycf2* gene contained the most, with 29, 24, 21 repeats in *S. eriocephalum*, *S. delavayi*, and *S. incisodentatum* K. T. Fu, respectively (Table S4).

We discovered 868 SSRs and the number of SSRs differed between the eleven *Seseli* plastomes, with *S. delavayi* having the fewest (66 SSRs) and *S. glabratum* having the most (90 SSRs). The number of Mono-, Di, Tri-, Tetra-, Penta- and Hexa- SSRs were 520, 189, 25, 103, 18, and 13, respectively (Fig. 3B, Table S5). Most of the SSRs were situated in LSC regions and intergenic spacers. However, the protein-coding gene *ycf1* contained the most abundant SSRs, with 48, and *ccsA* contained 20 SSRs (Table S5).

**Fig. 4:**
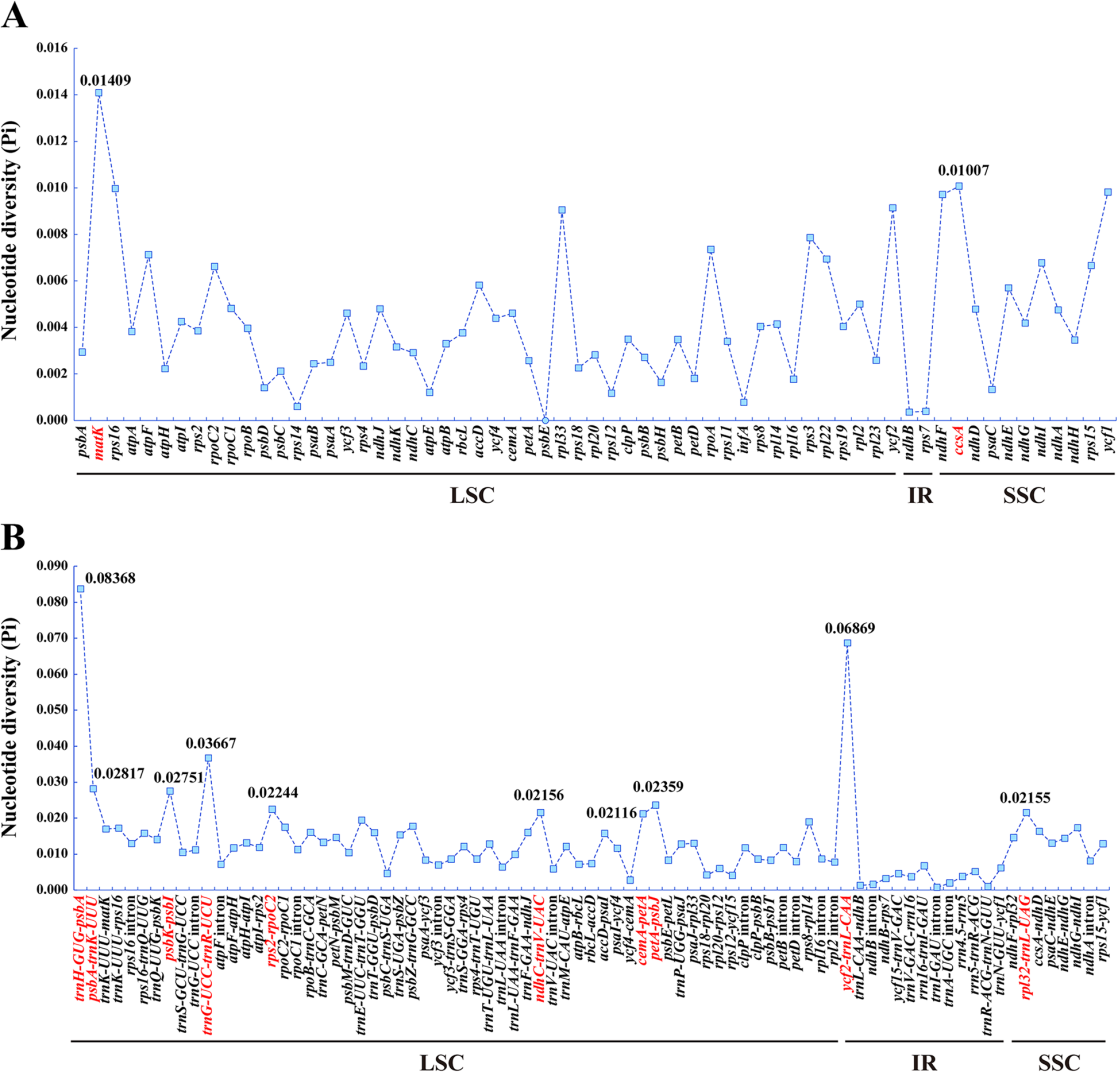
Comparative analysis of the nucleotide diversity (Pi) values among the eleven *Seseli* plastomes: (**A**) protein-coding genes, (**B**) non-coding and intron regions

We calculated the nucleotide diversity (Pi) of 63 protein-coding genes, 81 non-coding regions and introns in LSC, SSC, and IR (Fig. 4, Table S6). The Pi values of the gene regions ranged from 0 (*psbE* gene) to 0.01409 (*matK* gene) with a mean value of 0.00428, and the Pi values of the non-coding regions and introns ranged from 0.00077 (*trnI*-*GAU* intron) to 0.08368 (*trnH-GUG-psbA*) with a mean value of 0.01325 (Table S6). Two protein-coding genes (*matK* and *ccsA*) with relatively high nucleotide diversity (Pi > 0.01) were detected, while ten non-coding regions and introns with high nucleotide diversity (Pi > 0.02) were detected, namely *trnH-GUG*-*psbA*, *ycf2*-*trnL-CAA*, *trnG-UCC*-*trnR-UCU*, *psbA*-*trnK-UUU*, *psbK-psbI*, *petA*-*psbJ*, *rps2*-*rpoC2*, *ndhC*-*trnV-UAC*, *rpl32*-*trnL-UAG* and *cemA*-*petA* (Fig. 4, Table S6). These mutation hotspot regions were selected as candidate DNA barcodes.

### Phylogenetic analyses

**Fig. 5:**
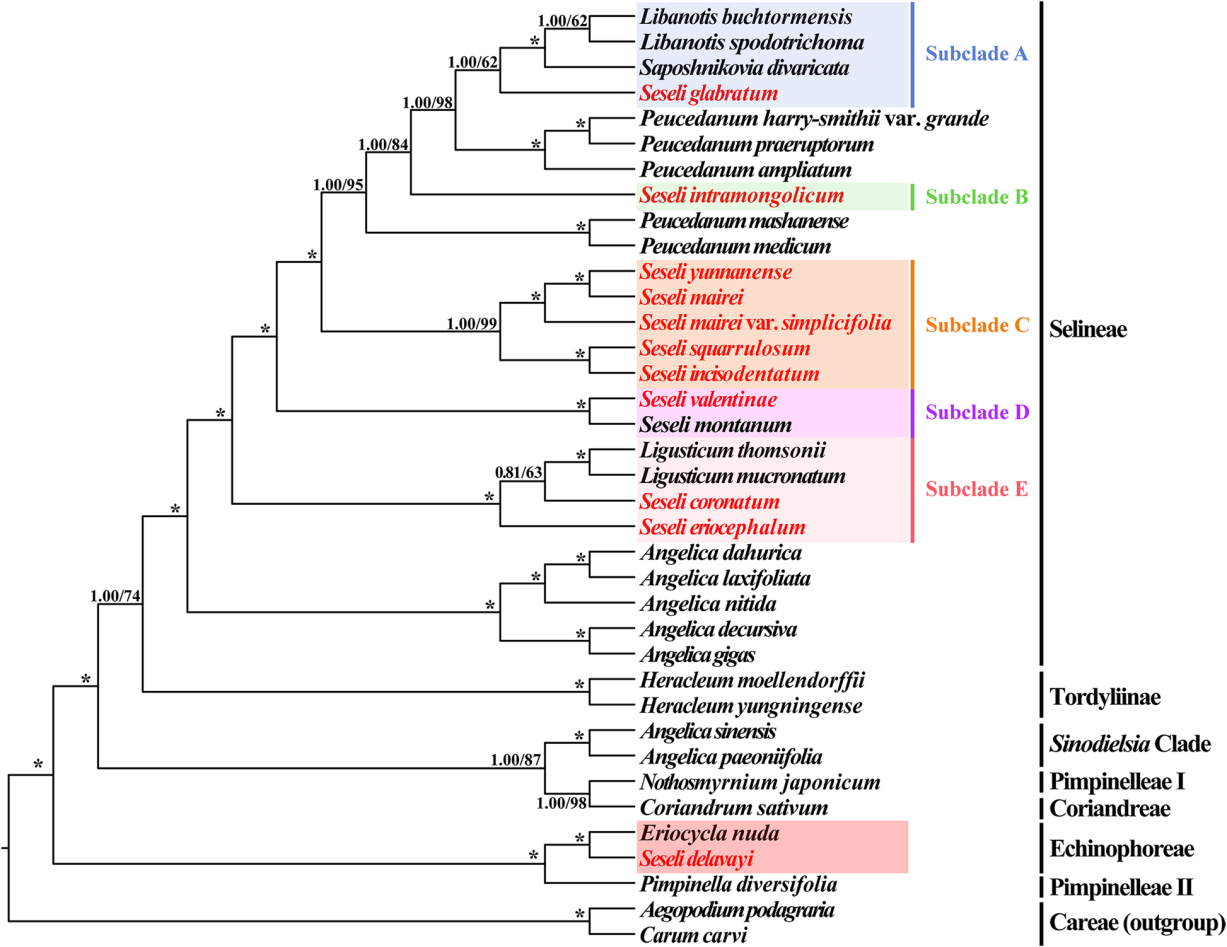
The plastome CDS-based phylogenetic tree constructed by Bayesian inference (BI) and maximum likelihood (ML) with the posterior probabilities of BI and the bootstrap values of ML above the branches, respectively, (*) represents maximum support in both two analyses

Thirty-seven plastome CDS and 59 nrDNA sequences, the two supermatrices, were used to reconstruct the phylogeny of *Seseli*, respectively. We found several incongruences in topologies between CDS-based and nrDNA-based phylogenetic trees. Nevertheless, the topologies indicated that the *Seseli* taxa fell into two tribes (Selineae and Echinophoreae) and were not clustered as a monophyletic group (Fig. 5, Fig. S1). Two types of support values: Bayesian inference (BI) posterior probabilities (PP) and ML bootstrap values (BS) were shown on the phylogenetic trees.

For CDS-based phylogenetic trees, the ML and BI trees were highly consistent in topology (Fig. 5). *S. delavayi* clustered with *Eriocycla nuda*, belonging to Echinophoreae, with strong support (PP = 1.00, BS = 100) and it was far from the main branches of *Seseli*. Whereas other *Seseli* taxa were members of Selineae (Fig. 5). In Selineae, the *Seseli* taxa failed to cluster in a clade, but were divided into five subclades (Fig. 5). *S. glabratum* clustered with *Libanotis buchtormensis*, *Libanotis spodotrichoma* K. T. Fu, and *Saposhnikovia divaricata* (Turcz. ex Ledeb.) Schischk. with relatively high support in BI (PP = 1.00) but the support in ML was rather weak (BS = 62), belonging to Subclade A. *S. intramongolicum* Ma formed a single Subclade B and diverged from Subclade A + *Peucedanum* subclade [*Peucedanum harry-smithii* var. *grande* (K.T.Fu) Shan et Sheh + *Peucedanum praeruptorum* Dunn + *Peucedanum ampliatum* K.T.Fu] with high support (PP = 1.00, BS = 98). *S. yunnanense*, *S. mairei*, *S. mairei* var. *simplicifolia*, *S. squarrulosum*, and *S. incisodentatum* formed a robust Subclade C (PP = 1.00, BS = 99), in which *S. yunnanense* + *S. mairei*, clustered with *S. mairei* var. *simplicifolia* with strong support (PP = 1.00, BS = 100), and *S. squarrulosum* and *S. incisodentatum* clustered together (PP = 1.00, BS = 100). *S. valentinae* and *Seseli montanum* L. formed a robust Subclade D (PP = 1.00, BS = 100). *Seseli coronatum* Ledeb. and *S. eriocephalum* were in Subclade E, in which *S. coronatum* was more closely related to the *Ligusticum* subclade (*Ligusticum thomsonii* + *Ligusticum mucronatum*) but with moderate support (PP = 0.81, BS = 63), and *S. eriocephalum* was located at the base of Subclade E with strong support (PP = 1.00, BS = 100).

The concatenated nrDNA dataset included 984 aligned characters. The ML and BI trees were nearly identical in topology, and several branches with quite low support (< 50% bootstrap support) were treated as parallel branches (Fig. S1). *S. delavayi* was also far from the other *Seseli* taxa and clustered with *Eriocycla nuda* within the tribe Echinophoreae with strong support (PP = 1.00, BS = 100). The other *Seseli* taxa belonged to Selineae. Nine *Seseli* taxa, being *S. arenarium* M.Bieb., *S. hartvigii* Parolly & Nordt, *S. andronakii* Woronow ex Schischk., *S. grandivittatum* (Sommier & Levier) Schischk., *S. serpentinum* B.L.Burtt ex H.Duman & E.Doğan, *S. alexeenkoi* Lipsky, *S. globiferum* Vis., *S. leptocladum* Woronow, and *S. tortuosum* formed the robust “*S. tortuosum*” clade (PP = 1.00, BS = 95). Eight *Seseli* taxa, being *S. corymbosum* Boiss. & Heldr., *S. paphlagonicum* Pimenov & Kljuykov, *S. lehmannii* Degen, *S. ponticum* Lipsky, *S. rupicola* Woronow, *S. resinosum* Freyn & Sint., *S. dichotomum* Pall. ex M.Bieb., and *S. gummiferum* Pall. ex Sm. formed the robust “*S. gummiferum*” clade with high support in BI (PP = 1.00) but weak support in ML (BS = 67). Five *Peucedanum* taxa and *Saposhnikovia divaricata* formed a clade (PP = 1.00, BS = 64). Then, these three clades clustered together with weak support (PP = 0.72, BS = 31). *S. yunnanense*, *S. mairei*, *S. mairei* var. *simplicifolia*, *S. squarrulosum*, and *S. incisodentatum* formed a robust clade (PP = 1.00, BS = 98). *S. coronatum* clustered with *S. glabratum* (PP = 0.83, BS = 62). *S. eriocephalum* formed a single branch. *S. transcaucasicum* (Schischk.) Pimenov & Sdobnina clustered with *Libanotis sibirica* (L.) C. A. Mey. (PP = 1.00, BS = 100), while *S. intramongolicum* clustered with *S. marashicum* E.Doğan & H.Duman (PP = 1.00, BS = 100), then, the two subclades clustered together. *S. valentinae* and *Ligusticum mucronatum* formed a clade (PP = 0.86, BS = 58).

### Comparative plastome analyses

**Fig. 6:**
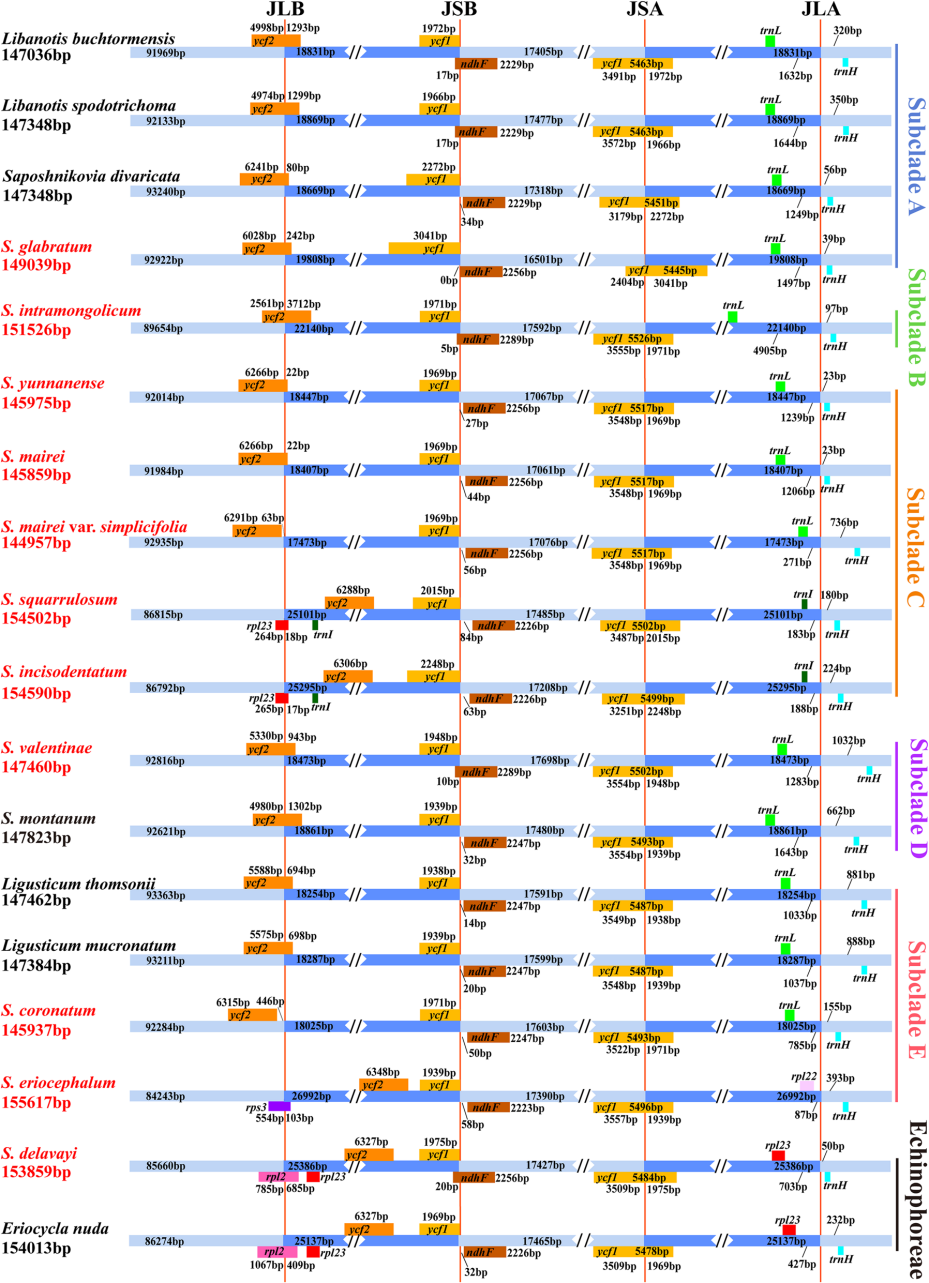
Comparison of the borders of LSC, SSC, and IR regions among the eighteen plastomes. Different boxes for genes represent the gene position

We investigated the plastome structural differences, mainly focusing on the borders of LSC/IRb (JLB), IRb/SSC (JSB), SSC/IRa (JSA), and IRa/LSC (JLA) (Fig. 6). Taxa within Subclade A had relatively similar structures, in which *S. glabratum* had the shortest SSC region (16,501 bp) and the longest *ycf1* gene extended into the IRa region (3,041 bp) among the eighteen plastomes. Subclade B: *S. intramongolicum* had the longest part of *ycf2* gene extended into the IRb region (3,712 bp) and an extreme distance of *trnL-CAA* gene from the JSA (4,905 bp) among the eighteen plastomes. Subclade C: *S. yunnanense* and *S. mairei* had highly similar structures, while *S. mairei* var. *simplicifolia* had 63 bp between the *ycf2* gene and the JLB. *S. squarrulosum* and *S. incisodentatum* had similar structures: the JLB was within the *rpl23* gene and the JLA was located between the *trnI-CAU* and *trnH-GUG* gene. Subclade D: *S. valentinae* and *S. montanum* had relatively similar structures, in which *S. valentinae* had the farthest distance between the *trnH-GUG* gene and the JLA (1,032 bp) among the eighteen plastomes. Subclade E: *Ligusticum thomsonii* and *Ligusticum mucronatum* had similar structures, the *ycf2* gene of *S. coronatum* was distant from the JLB (446 bp), while the JLB of *S. eriocephalum* was within the *rps3* gene and the JLA located between the *rpl22* and *trnH-GUG* gene. Echinophoreae: *S. delavayi* and *Eriocycla nuda* had the JLB within the *rpl2* gene and the JLA located between the *rpl23* and *trnH-GUG* gene (Fig. 6).

**Fig. 7:**
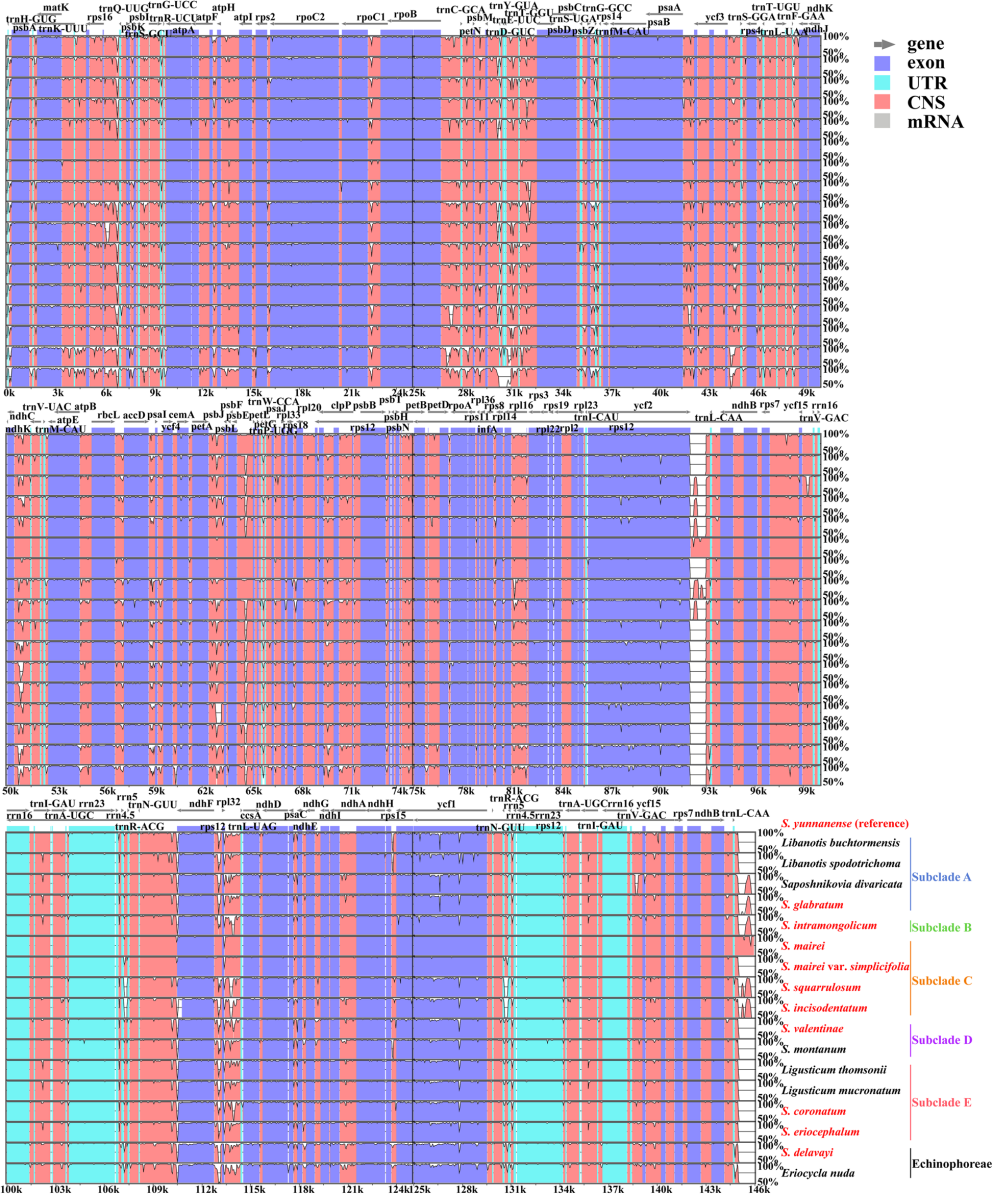
mVISTA-based sequence identity plots for the eighteen plastomes with *S. yunnanense* as the reference

The eighteen plastome sequence divergence analysis suggested that the coding regions were more conserved than the non-coding regions, and the IR regions were more conserved than the single copy regions (Fig. 7). Taxa within different subclades varied widely in some regions (e.g., *rps16*-*trnQ-UUG*, *rpoB*-*trnC-GCA*, *petA*-*psbJ*, *rpl32*-*trnL-UAG*, *ycf1*, *ycf2*), while the sequence divergence of taxa within the same subclade was relatively low.

**Fig. 8:**
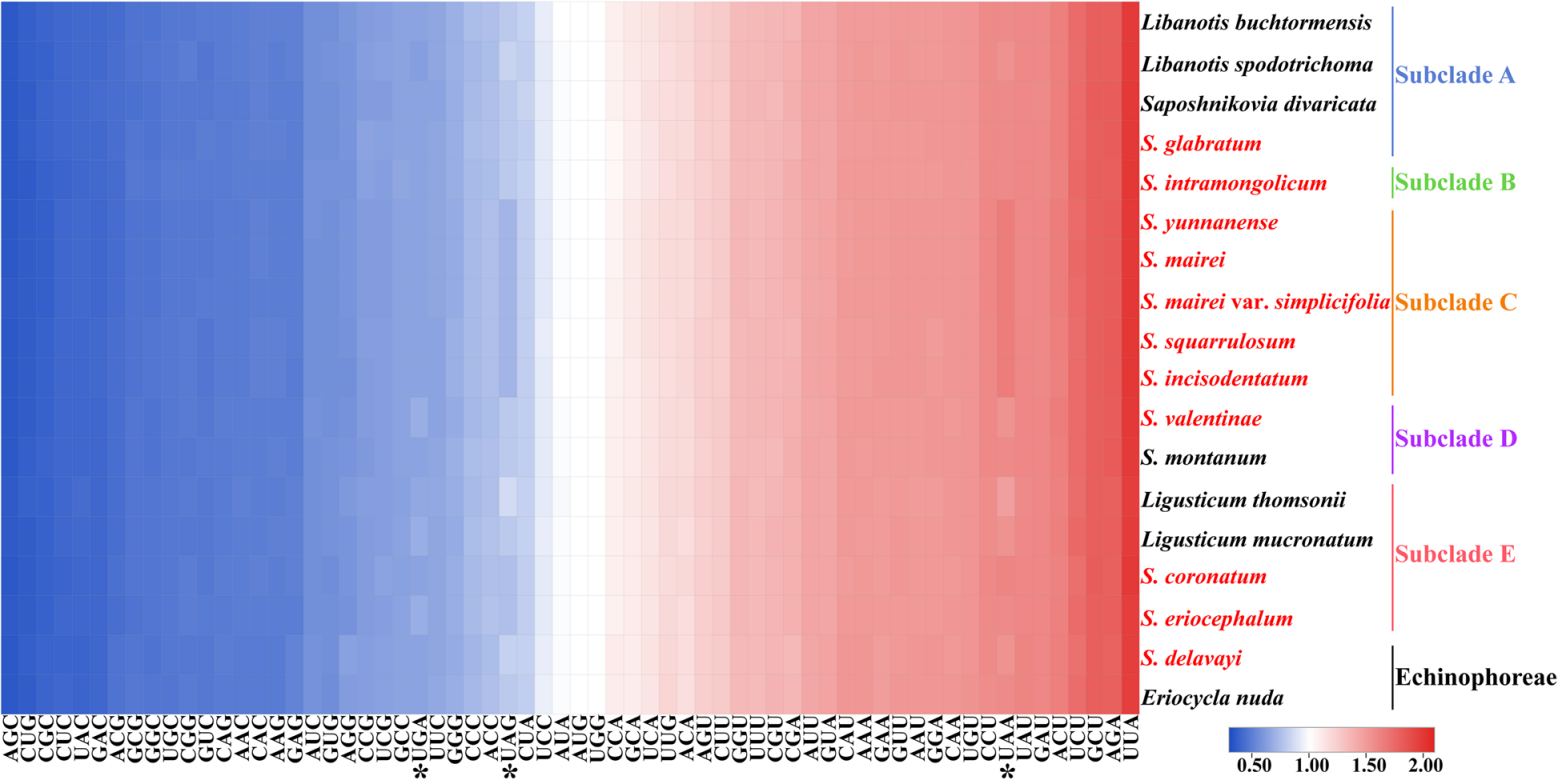
The RSCU values of 80 protein-coding genes for the eighteen plastomes. (*) to denote the terminator codons

The length of the 80 common CDS of the eighteen plastomes ranged from 68,148 bp in *Libanotis buchtormensis* to 68,313 bp in *S. eriocephalum* (Table S7). Codons encoding leucine (Leu) had the most (2,399–2,426), while codons encoding cysteine (Cys) had the fewest (235–243). The RSCU values of all codons ranged from 0.34 to 2.00 (Fig. 8, Table S7). The heatmap showed that 30 types of codons were used more frequently (i.e., RSCU value > 1) and ended with a purine (A/U) except for UUG. Considering the three types of terminator codons (UGA, UAG, and UAA), taxa that belonged to Subclade C (*S. yunnanense*, *S. mairei*, *S. mairei* var. *simplicifolia*, *S. squarrulosum*, and *S. incisodentatum*) had lower RSCU values of UAG (RSCU = 0.71) and higher values of UAA (RSCU = 1.65) than others. Overall, usage of codons showed bias in different subclades, and similarity in different taxa within the same subclade (Fig. 8, Table S7).

### Mericarp morphology

**Fig. 9:**
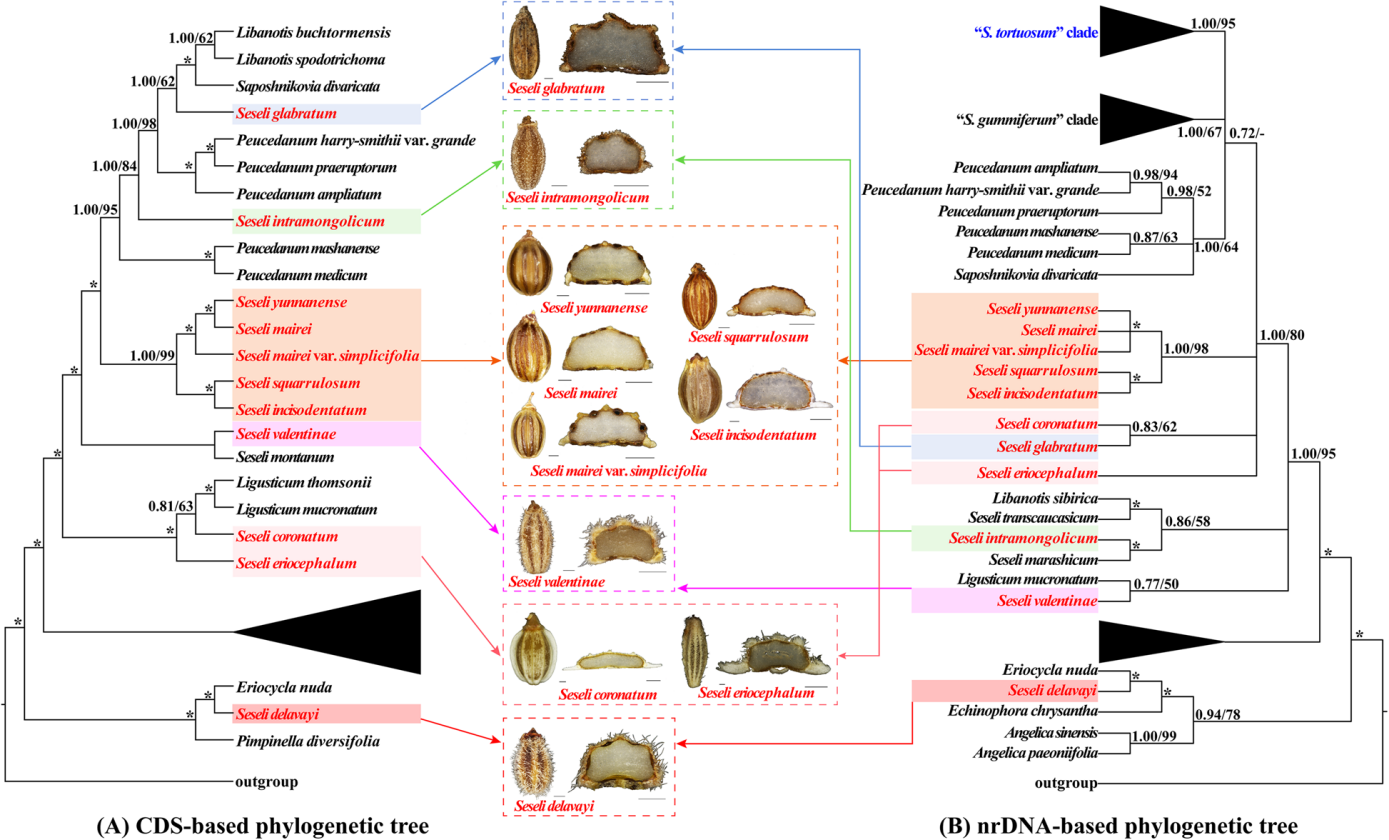
Mapping carpological characteristics to the two phylogenetic trees, different colors indicating correspondence. Scale bars: dorsal side views = 0.5 mm, transverse sections = 0.5 mm. (**A**) CDS-based phylogenetic tree, (**B**) nrDNA-based phylogenetic tree

We mapped the mericarps of the eleven *Seseli* taxa to the two phylogenetic trees (Fig. 9). *S. glabratum* in Subclade A, had mericarp elliptic or narrowly ovoid, slightly dorsally compressed, finely papillose or slightly scabrous, sometimes subglabrous, endosperm flat on commissural side, calyx teeth obsolete, ribs equal, prominent, filiform or shortly keeled, and vittae 1 in each furrow, 2 on commissure. *S. intramongolicum* in Subclade B, had mericarp oblong, slightly compressed dorsally to laterally, densely papillose-pubescent, endosperm flat on commissural side, calyx teeth small and triangular, all ribs equal, keeled and filiform, and vittae 1 in each furrow, 2 on commissure. Five taxa in Subclade C, had mericarps ovoid to elliptic, compressed dorsally, glabrous, endosperm flat on commissural side, and numerous vittae in commissure (2–10) and each furrow (1–4, not solitary). However, these five taxa were different in their ribs and calyx teeth: *S. yunnanense*, *S. mairei*, and *S. mairei* var. *simplicifolia* had ribs narrowly keeled or rounded, while *S. squarrulosum* and *S. incisodentatum* had median and lateral ribs keeled and filiform, marginal ribs winged. The first three taxa had calyx teeth obsolete, while *S. squarrulosum* had small calyx teeth and *S. incisodentatum* had broadly triangular calyx teeth. *S. valentinae* in Subclade D, had mericarp ovoid or oblong-ovoid, dorsally compressed, densely puberulent, endosperm flat on commissural side, calyx teeth small and triangular, all ribs equal, obtuse-keeled, and vittae 1 in each furrow, 2 on commissure. For Subclade E, *S. coronatum* had mericarp oblong, compressed dorsally, sparsely puberulent, endosperm flat on commissural side, calyx teeth obsolete, median and lateral ribs keeled and filiform, marginal ribs winged, and numerous vittae in commissure (8–12) and each furrow (3–5). *S. eriocephalum* had mericarp oblong, slightly dorsally compressed, densely tomentose, calyx teeth obsolete, ribs thick, marginal ribs slightly winged, and vittae 1 in each furrow, 2 on commissure. *S. delavayi* in Echinophoreae, had mericarps ovoid, slightly dorsally compressed, densely white hispid, endosperm slightly concave on commissural side, calyx teeth present and triangular, all ribs equal, keeled and filiform, hidden by indumentum, and vittae 1 in each furrow, 2 on commissure.

## Discussion

In this study, we newly generated eleven plastomes to investigate the plastome features and evolution of *Seseli*. The genome length, gene numbers, IR/SC borders, and repeat composition were variable, which further implied the complexity of plastomes evolution and the non-monophyly of *Seseli*. Then, we reconstructed the phylogeny of *Seseli* based on plastomes and nrDNA sequences. Both CDS-based and nrDNA-based phylogenetic trees indicated that the *Seseli* taxa did not form a monophyletic group, which was consistent with previous studies using molecular fragments [15–21]. The eleven newly sequenced *Seseli* taxa did not cluster with *S. tortuosum*, in which *S. delavayi* clustered with *Eriocycla* belonging to Echinophoreae and the others belonging to Selineae. The comparative plastome analyses and morphological characteristics confirmed the reliability of the phylogenetic analyses and implied the complex evolution of *Seseli*. We suggest that “a narrow sense” of *Seseli* is meaningful for further study and the current taxonomic system of *Seseli* needs to be revised.

### Plastome features and evolution

We observed that the genome structure (the typically quadripartite structure) and GC content of these eleven plastomes were conserved, and there was no gene rearrangement or loss among these eleven plastomes. These conservations are commonly in other genera of Apiaceae [28, 29], which might be related to stable plastome function. However, the genome length (144,957 bp-155,617 bp), gene numbers (128–134), IR/SC borders, and repeat composition were variable, which might imply the complexity of plastomes evolution and the non-monophyly of *Seseli*. In addition, twelve mutation hotspot regions (*matK* gene, *ccsA* gene, *trnH-GUG*-*psbA*, *ycf2*-*trnL-CAA*, *trnG-UCC*-*trnR-UCU*, *psbA*-*trnK-UUU*, *psbK-psbI*, *petA*-*psbJ*, *rps2*-*rpoC2*, *ndhC*-*trnV-UAC*, *rpl32*-*trnL-UAG* and *cemA*-*petA*) longer than 200 bp with high Pi values were selected as candidate DNA barcodes for phylogenetic analysis and species identification of *Seseli*. Among them, *matK* gene, *ccsA* gene, and *trnH*-*psbA* region have been used as universal DNA barcodes in studying plant phylogeny [39–41]. We will further explore the reliability and effectiveness of these regions in future studies.

#### A suggestion of “a narrow sense” for *Seseli* based on phylogeny and morphology

In the nrDNA-based phylogenetic tree, the *Seseli* taxa did not form a monophyletic group and were divided into several branches. The “*S. tortuosum*” clade and the “*S. gummiferum*” clade were separated with high support, which was consistent with a previous study [42]. However, these two clades were closer to the clade consisting of five *Peucedanum* taxa and a monotype genus, *Saposhnikovia* Schischk., while they did not cluster with *S. transcaucasicum*, *S. marashicum*, and the eleven newly sequenced *Seseli* taxa. After determining the morphological characteristics of the taxa belonging to the “*S. tortuosum*” and “*S. gummiferum*” clade (Table S8), we found that the nine *Seseli* taxa belonging to the “*S. tortuosum*” clade shared many common characteristics: the segments of basal leaves linear to lanceolate, bracts nearly absent, bracteoles linear to lanceolate, the number of rays no more than 20 (except *S. globiferum*) and unequal, calyx teeth very minute, mericarps ovoid or oblong, and ribs prominent. According to the literature and the results of Lyskov et al*.* [42], taxa belonging to the “*S. gummiferum*” clade were morphologically different from the “*S. tortuosum*” clade (e.g., *S. gummiferum* and *S. lehmannii* had several bracts, *S. rupicola* had conspicuous calyx teeth, *S. corymbosum* had 30–70 rays) (Table S8). In addition, taxa of the two clades differ geographically: taxa belonging to the “*S. tortuosum*” clade are mainly distributed on the northern coast of the Mediterranean, while taxa belonging to the “*S. gummiferum*” clade are mainly distributed near the Black Sea. The nrDNA-based phylogenetic tree had several parallel branches, which resulted in lack of resolution for interspecific relationships, but it was still clear that *Seseli* was non-monophyly.

Most *Seseli* species are regional endemics and the composition of the type section of *Seseli* (i.e., the species closely related to the type species *S. tortuosum* L.) has not been clearly defined, which leads to imperfect and incomplete classification among *Seseli* species [42]. Moreover, given that the genera (*Peucedanum*, *Ligusticum*, and *Libanotis*) related to *Seseli* were also non-monophyly [16, 17, 28, 30], which made studying the phylogeny and taxonomy of *Seseli* harder. It is worth noting that many phylogenetic and taxonomic problems of *Peucedanum* and *Ligusticum* have been effectively resolved after considering a narrower sense [15, 43]. Overall, to further investigate the phylogeny and taxonomy of *Seseli*, we suggest that it is important to treat *Seseli* in a narrow sense. Thus, according to our results, the “*S. tortuosum*” clade could be referred to as the narrow sense of *Seseli* (within Selineae), containing common features such as leaf segments linear to lanceolate, bracts nearly absent, bracteoles linear to lanceolate, rays unequal, calyx teeth very minute, mericarps ovoid or oblong, ribs prominent, and consisting of the following species: *S. tortuosum*, *S. arenarium*, *S. hartvigii*, *S. andronakii*, *S. grandivittatum*, *S. serpentinum*, *S. alexeenkoi*, *S. globiferum*, and *S. leptocladum*.

#### The phylogenetic position of *S. delavayi* and the taxonomic relationship between *Seseli* and *Eriocycla*

Previous studies have revealed that members belonging to *Seseli* are distributed into three tribes: Selineae, Pimpinelleae, and Apieae [17]. However, *S. diffusum* belonging to Pimpinelleae has been treated as *Psammogeton difusum* (Roxb ex Sm.) Rech.f. ex Pimenov [20], while *S. webbii* belonging to Apieae has been treated as *Canaria tortuosa* (Webb & Berthelot) Jim.-Mejías & P.Vargas [21]. Consequently, the remaining *Seseli* taxa still belong to Selineae. However, in our study, we found that *S. delavayi* was furthest from the main branches of *Seseli* (within Selineae), but clustered with *Eriocycla nuda* and belonged to Echinophoreae [4]. *S. delavayi* had a similar plastome structure and SC/IR borders to *Eriocycla nuda*. It also possessed the most different morphological characteristics from other *Seseli* taxa but is similar to *Eriocycla* species: pubescent throughout, peduncles elongate, bracts 5-7, bracteoles longer than 2 × pedicels, prominent calyx teeth, and fruit densely white hispid (Table S9).

Taxonomic controversies between *Seseli* and *Eriocycla* have always existed: Degtjareva [12] proposed that several *Seseli* species (e.g., *S. delavay*i, *S. afghanicum*) should be transferred into *Eriocycla*, while Pimenov [8] treated *Eriocycla* taxa as synonyms of *Seseli* taxa based on type specimens. In our study, it was clear that the type species of *Seseli*, *S. tortuosum*, was located in Selineae, while the type species of *Eriocycla*, *Eriocycla nuda*, was nested in Echinophoreae. Additionally, *S. tortuosum* and *Eriocycla nuda* were morphologically dissimilar (base with clothed in fibrous remnant sheaths vs. base without clothed in fibrous remnant sheaths, peduncles not elongate vs. peduncles elongate, bracts absent or 0-1 vs. bracts 3-5, petals white-violet vs. petals bright yellow) [5, 44]. Therefore, *Eriocycla* should be identified as a separate genus based on our molecular and morphological evidence. Additionally, introducing more *Eriocycla* taxa would be useful to establish a robust phylogenetic framework for *Eriocycla* and resolve the taxonomic problem of *S. delavayi*.

#### The taxonomic complexity of *Seseli* based on phylogeny, comparative plastome analyses and morphological characteristics

*Seseli* taxa within Selineae failed to form a monophyletic group but were divided into five subclades. The comparative plastome analyses and morphological characteristics confirmed the reliability of our phylogenetic analyses and implied the complex evolution of *Seseli*. For the comparative plastome analyses, *Seseli* taxa belonging to different subclades showed distinguishing SC/IR borders, sequence divergence, and codon usage, which implied the complexity of plastome evolution and the non-monophyly of *Seseli*. Additionally, *Seseli* taxa had significantly different morphological features. For example, *S. yunnanense*, *S. mairei*, *S. mairei* var. *simplicifolia*, *S. squarrulosum*, and *S. incisodentatum* had glabrous mericarps, while other *Seseli* taxa had more or less hairy mericarps: *S. glabratum* had sparsely papillose, *S. intramongolicum* had densely papillose-pubescent, *S. valentinae* had densely puberulent, *S. coronatum* had sparsely puberulent, and *S. eriocephalum* had densely tomentose. In addition, mericarp in transverse section was slightly dorsally to laterally compressed in *S. intramongolicum* but strongly dorsally compressed in *S. coronatum*. Even though these taxa had different morphological characteristics, they also possessed several common characteristics: bracts absent or 1-2, bracteoles numerous, calyx teeth almost obsolete, and mericarp ovoid or oblong, which were very important taxonomic characteristics of *Seseli* [45]. These morphological similarities and differences of taxa indicated that *Seseli* was indeed a taxonomically complex genus.

The incongruences between nrDNA-based and plastome-based phylogenetic trees often appear in plant phylogenetic analyses [28, 46–48], and there is no exception in our results. For example, *S. glabratum* clustered with *Libanotis buchtormensis*, *Libanotis spodotrichoma*, and *Saposhnikovia divaricata* in the CDS-based phylogenetic tree, while it clustered with *S. coronatum* in the nrDNA-based phylogenetic tree, *S. valentinae* clustered with *S. montanum* in the CDS-based phylogenetic tree, while it was clustered with *Ligusticum mucronatum* in the nrDNA-based phylogenetic tree. These incongruences might be the result of hybridization, introgression, and incomplete lineage sorting (ILS) [49]. Moreover, Wen [50] proposed that chloroplast capture events in Apiaceae induced by early hybridization explained the incongruence of positions between tribes in the two phylogenetic trees. Further study is needed to identify the cause of the nuclear-plastome conflict in *Seseli*.

It is impossible for us to conduct taxonomic treatments of several *Seseli* taxa due to the lack of adequate morphological and molecular data of *S. tortuosum*. We recommend that comprehensive studies of morphological characteristics and molecular phylogeny should reduce the uncertainties in the taxonomy of *Seseli*. Overall, in our study, we verify the non-monophyly of *Seseli* based on both plastomes and nrDNA sequences, and this provides a foundation for studying the evolution, phylogeny, and taxonomy of *Seseli*.

## Conclusion

In this study, we newly sequenced, assembled and annotated complete plastomes of eleven *Seseli* taxa. We observed that the genome length, gene numbers, IR/SC borders, and repeat composition of the *Seseli* plastomes were variable. Several appropriate mutation hotspot regions might be developed as candidate DNA barcodes for evolution, phylogeny, and species identification of *Seseli*. Thirty-seven plastome CDS and 59 nrDNA sequences were used to perform the phylogenetic analysis of *Seseli*. The phylogenetic results identified that *Seseli* was not a monophyletic group. Moreover, the eleven newly sequenced *Seseli* taxa did not cluster with *S. tortuosum* (the type species of *Seseli*, belonging to the tribe Selineae), where *S. delavayi* clustered with *Eriocycla* belonging to Echinophoreae and the other ten belonged to Selineae. The comparative plastome and morphological characteristics analyses confirmed the reliability of the phylogenetic analyses and implied the complex evolution of *Seseli*. We suggest that “a narrow sense” of *Seseli* will be meaningful for further study and the current taxonomic system of *Seseli* needs to be revised. Overall, our study can provide new insights into the phylogenetic relationships and taxonomic framework of *Seseli*.

## Methods

### Sample collection

Fresh and mature green leaves from adult plants of eleven taxa, namely *S. mairei*, *S. mairei* var. *simplicifolia*, *S. yunnanense*, *S. squarrulosum*, *S. incisodentatum*, *S. glabratum*, *S. intramongolicum*, *S. valentinae*, *S. coronatum*, *S. eriocephalum*, and *S. delavayi*, were collected from the wild (including Xinjiang, Gansu, Ningxia, and Yunnan provinces) and immediately dried with silica gel for subsequent treatment. These taxa belong to four of the five sections (*Sect. Seseli*, *Sect. Hippomarathroidea DC.*, *Sect. Macrostylopodium Schischk.*, and *Sect. Pseudosilaus Schischk.*) according to the treatment of *Flora Republicae Popularis Sinicae* (FRPS) [51]. The formal identification of these eleven samples was undertaken by Associate Professor Songdong Zhou (Sichuan University). Voucher specimens of the above taxa were deposited in the herbarium of Sichuan University (SZ) (Table S1).

### DNA extraction, sequencing, assembly and annotation

Total genomic DNA was extracted from silica gel-dried materials using the modified CTAB method [52]. Then, we amplified ITS and ETS (internal and external transcribed spacer) sequences of these eleven *Seseli* taxa with primers ITS-4 (5’-TCCTCCGCTTATTGATATGC-3’), ITS-5 (5’-GGAAGTAAAAGTCGTAACAAGG-3’) [53], 18S-ETS (5’-ACTTACACATGCATGGCTTAATCT-3’) [54], and Umb-ETS (5’-GCGCATGAGTGGTGAWTKGTA-3’) [55]. Polymerase chain reactions (PCRs) were performed in a 30 µL volume with 2 µL plant total DNA, 1.5 µL forward primer,1.5 µL reverse primer, 15 µL volume 2 × Taq MasterMix (cwbio, Beijing, China), and 10µL ddH_2_O. We used the software DNAstar-SeqMan to edit and obtain the newly assembled ITS and ETS sequences [56].

For plastomes, raw data of the eleven newly sequenced *Seseli* taxa were generated by Illumina platform, generating 150 bp paired-end reads at Novogene (Beijing, China). The raw data was filtered through fastP v0.15.0 (-n 10 and -q 15) to ensure high quality [57]. After quality control, we acquired at least 5 GB of clean reads for each taxon. The clean reads were assembled using the program NOVOPlasty v2.6.2 [58], with default parameters and the *rbcL* gene sequence of *S. tortuosum* (MW662022) as the seed sequence. Genome annotation was performed using Plastid Genome Annotator (PGA) [59] with *S. montanum* (KM035851) as the reference. Manual adjustment compared with related species’ plastomes was conducted in Geneious v9.0.2 [60]. Then, we used the online program Organellar Genome DRAW (OGDRAW) [61] to draw circular plastome maps. Additionally, the gene rearrangements among the eleven *Seseli* plastomes were detected using Mauve Alignment [62] in Geneious v9.0.2 [60].

The newly generated plastomes, ITS and ETS sequences of the eleven *Seseli* taxa have been submitted to the GenBank under accession numbers ON975056-ON975066, ON980800- ON980810, and ON980787-ON980797 (Table S2).

### Repeat sequence and nucleotide diversity analyses

The online REPuter program [63] was used to identify repeat sequences, and four types were included: forward, palindromic, reverse, and complementary repeats. The parameter settings were as follows: (1) a minimum repeat size of 30 bp; (2) more than 90% sequence identity between two repeats; and (3) Hamming distance = 3. In addition, we used the Perl script MISA (http://pgrc.ipk-gatersleben.de/misa/sleben.de/misa/) to detect simple sequence repeats (SSRs) of the eleven *Seseli* plastomes with thresholds (the minimum number of SSRs) of 10, 5, 4, 3, 3, and 3, for mono-, di-, tri-, tetra-, penta-, and hexanucleotide SSRs, respectively.

DnaSP version 6.12.03 [64] was used to calculate the nucleotide diversity (Pi) of protein-coding genes, non-coding regions, and introns to identify mutation hotspot regions. To develop potential and useful molecular markers for future analyses, the length of regions we selected was longer than 200 bp [65].

### Phylogenetic analyses

Previous studies have shown that *Seseli* species are divided into three tribes: Selineae, Pimpinelleae, and Apieae [17]. Thus, in our study, we chose *Aegopodium podagraria* L. and *Carum carvi* L. belonging to the tribe Careae as the outgroup to root the phylogenetic tree, according to the results of Wen *et al.* [50]. The names of these tribes were mainly based on the work of Lyskov *et al.* [4] and Wen *et al.* [50]. All taxa and their accession numbers in GenBank included in the phylogenetic analysis are listed in Table S2.

Eighty common CDS of 37 Apiaceae taxa were extracted, respectively aligned, and concatenated as the plastome CDS dataset, using PhyloSuite v1.2.2 [66]. Previous studies of Apiaceae indicated that the nrDNA ITS, in conjunction with the ETS region, can provide more informative variation for phylogenetic reconstruction and allow for better resolution of relationships [55, 67, 68]. Thus, ITS and ETS sequences of 59 Apiaceae taxa were first respectively aligned using MAFFT v7.308 [69] and concatenated as the nrDNA dataset in PhyloSuite v1.2.2 [66]. Then, the two datasets (plastome CDS and nrDNA) were used to reconstruct the phylogeny of *Seseli*.

Maximum likelihood (ML) analysis was conducted using RAxML v8.2.8 [70] based on the best-fit GTRGAMMA model and 1000 bootstrap replicates. Bayesian inference was performed using MrBayes v3.2.7 [71] after the program Modeltest v3.7 [72] calculated the best-fitting models of nucleotide substitutions under the Akaike information criterion (AIC), and the best-fitting models were GTR+I+G for both nrDNA and plastome CDS dataset. Four independent Markov chains were run for 10,000,000 generations, with one tree sampled every 1,000 generations. The first 25% of the trees were discarded as burn-in. FigTree v1.4.2 [73] was used to edit the phylogenetic trees, with nodes under 50% bootstrap support being treated as parallel branches.

### Comparative plastome analyses

Comparative plastome analyses were based on phylogenetic results, and a total of eighteen plastomes belonging to Selineae and Echinophoreae were selected. Of the eighteen, eleven were the newly generated *Seseli* plastomes from this study. The remaining seven plastomes were from published data (*Libanotis buchtormensis*, *Libanotis spodotrichoma*, *Saposhnikovia divaricata*, *S. montanum*, *Ligusticum thomsonii*, *Ligusticum mucronatum*, and *Eriocycla nuda*).

The online program IRscope [74] was used to display the borders between the inverted repeat (IR) and single copy (SC) regions, aiming to illustrate the structural differences in the eighteen plastomes. Then, sequence divergence of the eighteen plastomes was performed with the online program mVISTA in Shuffle-LAGAN mode [75], with *S. yunnanense* as the reference.

Eighty common single-copy coding sequences of the eighteen plastomes were extracted, and then codon usage analysis and relative synonymous codon usage (RSCU) values [76] were calculated using the CodonW v1.4.2 program [77]. TBtools [78] was used to make a heatmap to visualize the RSCU values.

### Morphological data

Mericarps of the eleven *Seseli* taxa were collected from the field and were then photographed using a stereomicroscope (Nikon SMZ25). The mericarp terminology followed Kljuykov et al. [79]. Morphological data of these *Seseli* taxa and other related taxa involved in this study was obtained during our field observation, consulting type specimens, and previous literature review.

## Supplementary Information

**Additional file 1: Figure S1. **The nrDNA-based phylogenetic tree constructed by Bayesian inference (BI) and maximum likelihood (ML) with the posterior probabilities of BI and the bootstrap values of ML above the branches, respectively, (*) represents maximum support in both two analyses, (-) represents those nodes not occurring in the ML strict consensus tree. The black triangle indicates the type species of *Seseli*, *S. tortuosum*.

**Additional file 2: Table S1. **Collection locality and voucher information of the eleven *Seseli* taxa.

**Additional file 3: Table S2. **GenBank accession numbers of DNA sequences used in this study. The accession numbers of DNA sequences from our lab were in bold.

**Additional file 4: Table S3. **Gene contents of the eleven *Seseli* plastomes.

**Additional file 5: Table S4. **The repeat sequences distribution in the eleven *Seseli* plastomes.

**Additional file 6: Table S5. **Simple sequence repeats (SSRs) distribution in the eleven *Seseli* plastomes.

**Additional file 7: Table S6. **Nucleotide diversity (Pi) of coding and non-coding regions.

**Additional file 8: Table S7. **Codon usage and relative synonymous codon usage (RSCU) values of protein-coding genes of the eighteen plastomes.

**Additional file 9: Table S8. **Morphological data of the taxa belonging to the “*S. tortuosum*” and “*S. gummiferum*” clade.

**Additional file 10: Table S9. **Morphological data of the eighteen taxa in Selineae and Echinophoreae involved in this study.

## Data Availability

The newly sequenced plastomes, ITS, and ETS sequences of the eleven *Seseli* taxa have been submitted into NCBI with accession numbers: ON975056- ON975066, ON980800- ON980810, and ON980787-ON980797, respectively.

## Abbreviations

CTAB: Cetyl trimethylammonium bromide
PCR: Polymerase chain reaction
ITS: Internal transcribed spacer
ETS: External transcribed spacer
CDS: Single-copy coding sequences
bp: Base pair
rRNA: Ribosomal RNA
tRNA: Transfer RNA
SSR: Simple sequence repeat
ML: Maximum Likelihood
BI: Bayesian inference
AIC: Akaike information criterion
BS: Bootstrap value
PP: Posterior probability
Pi: Nucleotide diversity
RSCU: Relative synonymous codon usage
IR: Inverted repeat
SC: Single copy
LSC: Large single copy
SSC: Small single copy
