# Supplementary figures and images for: The phylogeny of Seseli (Apiaceae, Apioideae): insights from molecular and morphological data
Source: BMC Plant Biol. 2022 Nov 16;22:534. doi: 10.1186/s12870-022-03919-9 (PMC9667662; doi:10.1186/s12870-022-03919-9)

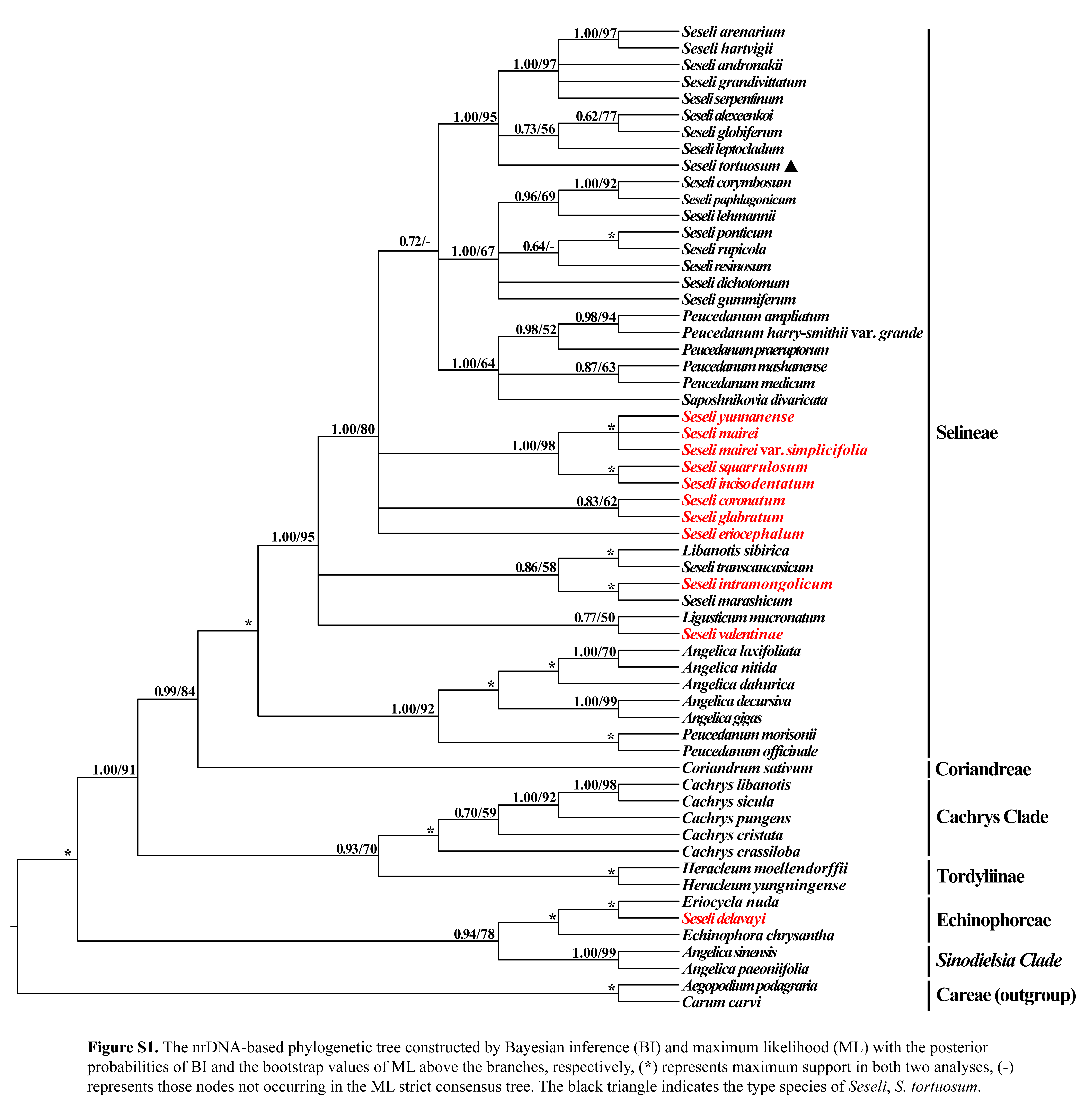

Supplement: Supplementary file 1 — Additional file 1: Figure S1. The nrDNA-based phylogenetic tree constructed by Bayesian inference (BI) and maximum likelihood (ML) with the posterior probabilities of BI and the bootstrap values of ML above the branches, respectively, (*) represents maximum support in both two analyses, (-) represents those nodes not occurring in the ML strict consensus tree. The black triangle indicates the type species of Seseli, S. tortuosum. [file 12870_2022_3919_MOESM1_ESM.tif]
